# Continuous Blood Pressure Indices During the First 72 Hours and Functional Outcome in Patients with Spontaneous Intracerebral Hemorrhage

**DOI:** 10.1007/s12028-024-02146-4

**Published:** 2024-10-25

**Authors:** Annerose Mengel, Vasileios Siokas, Rebecca Buesink, Sara Roesch, Kornelia Laichinger, Redina Ferizi, Efthimios Dardiotis, Jennifer Sartor-Pfeiffer, Constanze Single, Till-Karsten Hauser, Markus Krumbholz, Ulf Ziemann, Katharina Feil

**Affiliations:** 1 Department of Neurology and Stroke, Eberhard-Karls University of Tübingen, Tübingen, Germany; 2 Hertie Institute for Clinical Brain Research, Eberhard-Karls University of Tübingen, Tübingen, Germany; 3 Department of Neurology, Faculty of Medicine, School of Health Sciences, University Hospital of Larissa, University of Thessaly, Larissa, Greece; 4 Department of Neuroradiology, Eberhard-Karls University of Tübingen, Tübingen, Germany; 5 Department of Neurology, University Hospital of the Brandenburg Medical School, Rüdersdorf, Germany

**Keywords:** Intracerebral hemorrhage, Blood pressure variability, Functional outcome, Mortality

## Abstract

**Background:**

Management of intracerebral hemorrhage (ICH) is challenged by limited therapeutic options and a complex relationship between blood pressure (BP) dynamics, especially BP variability (BPV) and ICH outcome.

**Methods:**

In an exploratory analysis of prospectively collected data on consecutive patients with nontraumatic ICH between 2015 and 2020, continuous BP accessed via an arterial line extracted from the Intellispace Critical Care and Anesthesia information system (Philips Healthcare) was analyzed over the first 72 h post admission. Arterial lines were used as part of standard clinical practice in the intensive care, ensuring high fidelity and real-time data essential for acute care settings. BPV was assessed through successive variation (SV), standard deviation (SD), and coefficient of variation using all available BP measurements. Multivariate regression models were applied to evaluate the association between BPV indices and functional outcome at 3 months.

**Results:**

Among 261 patients (mean age 69.6 ± 15.2 years, 47.9% female, median National Institutes of Health Stroke Scale [NIHSS] score 6 [interquartile range 2–12]) analyzed, lower systolic BP upon admission (< 140 mm Hg) and lower systolic BPV were significantly associated with favorable outcome, whereas higher diastolic BPV correlated with improved outcomes. In the multivariate analysis, diastolic BPV (SD, SV) within the first 72 h post admission emerged as an independent predictor of good functional outcome (modified Rankin Scale score < 3; odds ratio 1.123, 95% confidence interval CI 1.008–1.184, *p* = 0.035), whereas systolic BPV (SD) showed a negative association. Patients with better outcomes also exhibited distinct clinical characteristics, including younger age, lower median NIHSS scores, and less prevalence of anticoagulation therapy upon admission.

**Conclusions:**

This study shows the prognostic value of BPV in the acute phase of ICH. Lower systolic BPV (SD) and higher diastolic BPV (SD, SV) were associated with better functional outcomes, challenging traditional BP management strategies. These findings might help to tailor a personalized BP management in ICH.

**Supplementary Information:**

The online version contains supplementary material available at 10.1007/s12028-024-02146-4.

## Introduction

Therapeutic options for intracerebral hemorrhage (ICH) remain limited, highlighting an urgent need for improved care approaches [1, 2]. The aging population and increasing use of anticoagulation will further lead to higher incidence of ICH [3–6].

Management of blood pressure (BP) is pivotal in ICH care given its established role in both the risk and progression of ICH [7]. Despite this recognition, there is no universally accepted strategy and consensus on optimal BP management in the acute phase following nontraumatic spontaneous ICH, reflecting the complex relationship between BP dynamics and ICH outcome [8]. Recent investigations into acute BP reduction have shown mixed effects on morbidity and mortality, suggesting a more complicated role between ICH progression and outcome and BP [9–12]. Traditionally, research has focused on systolic BP and specific time frames, often relying on intermittent, noncontinuous BP measurements [13]. The recent INTERACT-3 study, which proposed a comprehensive approach to acute management, including early intensive BP lowering, has provided further insights into optimizing ICH outcomes [14]. This approach may not fully capture the complexity of BP dynamics, especially in the acute phase of ICH. Blood pressure variability (BPV), defined as fluctuations in BP over a specific time period, is calculated using various measurements, including successive variation (SV), standard deviation (SD), coefficient of variation (CV), average real variability, and functional successive variation [15, 16]. Each of these approaches offers distinct insights into BPV, with implications for understanding the pathophysiological impact of BP dynamics on patient outcomes [17, 18].

Elevated systolic BPV has been linked to adverse outcomes in both acute ischaemic stroke and ICH, yet studies frequently neglect diastolic BP, mean arterial pressure (MAP), and the continuity of monitoring [19–27]. A recent study in the intensive care unit (ICU) setting showed that patients with stroke exhibited higher BPV compared to individuals without stroke and found increased in-hospital mortality and reduced likelihood of favorable discharge in conditions such as acute ischaemic stroke, ICH, and subarachnoid hemorrhage, suggesting BPV as an indicator of poor outcomes, possibly reflecting central autonomic network damage or the consequences of targeted BP management [28].

In addressing the gaps in current research, our study hypothesizes that patients with ICH with good functional outcomes (modified Rankin Scale [mRS] score < 3) will display decreased BPV relative to those with worse outcomes, extending our analysis to encompass diastolic BP and MAP through continuous arterial line monitoring over the initial 72 h after admission. Using big data analytics, we aim to thoroughly investigate BP and BPV’s role within a well-described ICH patient cohort. Through this prospective observational cohort study, we aim to comprehensively evaluate the prognostic significance of BP fluctuations on ICH patient outcomes. This approach seeks to elucidate BPV’s comprehensive impact, fostering the development of more tailored and personalized management strategies in acute ICH care.

## Methods

### Study Design

The current study is an observational study, with prospectively collected BP data within a local stroke registry. Although this study uses prospectively collected data, it is inherently retrospective because it analyzes preexisting data without a predefined protocol prior to patient follow-up.

### Study Population and Exclusion Criteria

During the present study, we collected data from consecutive patients diagnosed with spontaneous nontraumatic ICH admitted to the ICU and/or intermediate care of the University Hospital of Tübingen between December 1, 2015, and December 31, 2020. Data were extracted from our local stroke registry.

The following exclusion criteria were applied: (1) duration of hospital stay < 24 h, (2) neurosurgical intervention immediately after baseline computed tomography (CT), (3) traumatic ICH, (4) intracranial tumor as the etiology for ICH, (5) hemorrhagic transformation of ischaemic stroke, (6) patients with withdrawal of medical management at admission or within the first 24 h, and (7) incomplete data on functional outcome at 3 months’ follow-up.

All patients were treated using a standardized stepwise BP management protocol that has been established at our institution since 2012. This includes the intravenous administration of urapidil, clonidine, and Nepressol for systolic BP greater than 140 mm Hg. Nifedipine and nitroglycerin are used as reserve medications.

Patients were stratified according to the CLAS-ICH classification systems. The CLAS-ICH criteria classify hemorrhages based on location, size, and age, offering insights into the hemorrhage characteristics that could influence patient outcomes [29].

Patients’ clinical characteristics were extracted from the medical records. Functional outcome was assessed using the mRS either by telephone calls or by outpatient visits. Clinical outcome was assumed as excellent if the mRS score was 0–1 and good if the mRS score was 0–2 [30].

### Imaging Data

Each patient underwent initial CT imaging on admission, including noncontrast CT and CT angiography. Moreover, all participants underwent a follow-up brain imaging (either CT or magnetic resonance imaging) 24 h after admission. An independent experienced neuroradiologist assessed all images in a blinded and randomized fashion. White matter disease was identified according to the age-related white matter changes rating scale (Fazekas score) [31]. ICH volume was quantitatively assessed using the ABC/2 formula. Early hematoma expansion was defined either as absolute (≥ 6 mL) or relative (≥ 33%) change of ICH volume on follow-up brain imaging 24 h after admission compared to baseline imaging [32, 33].

### BP Measurement and Definition of BP Indices

Upon admission to the ICU, arterial lines were used for continuous BP monitoring as part of standard clinical practice in the neurological ICU, ensuring high fidelity and real-time data essential for acute care settings. These continuous BP recordings were systematically extracted from the clinical information system (Intellispace Critical Care and Anesthesia information system; Philips Healthcare) and used to assess BPV across various time intervals following admission for the first 72 h after admission. Specifically, absolute BP as well as BPV indices were evaluated for the intervals of 0–24 h, 0–48 h, and 0–72 h post admission as well as 24–48 h and 48–72 h, enabling a comprehensive analysis of BP dynamics during the first days of clinical care.

For each time interval, BPV was calculated using all available BP measurements, encompassing systolic BP, diastolic BP, and MAP. Time intervals of BP readings were every 5 min. The BPV indices calculated included SV, SD, and CV, providing a multidimensional view of BP fluctuation magnitude and frequency. The methodology for calculating each of these BPV indices is detailed in Table 1.

**Table 1:** Formulas for calculation of BPV

$$SV=\sqrt{\frac{1}{n-1}\sum_{i=1}^{n-1}{({X}_{i+1}-{X}_{i})}^{2}}$$
$$SD=\sqrt{(1/(n-1){\sum }_{(i=1)}^{(n-1)}{({BP}_{i}-{BP}_{\text{mean}})}^{2}}$$
CV = SD/mean × 100

BP, Blood pressure; BPV, Blood pressure variability; CV, Coefficient of variation; n, Number of values; SD, Standard deviation; SV, Successive variability

This approach ensured the use of the full spectrum of BP data, reflecting the intricate patterns of hemodynamic instability in the acute phase of ICH. Analysis was confined to patients with a complete set of BP data for the designated time frames, ensuring the integrity and reliability of the BPV assessment.

### Study End Points

The primary end point of this study was to assess the impact of various BPV indices on good outcome at follow-up after 90 days. Specifically, we focused on the SV, SD, and CV of systolic BP, diastolic BP, and MAP in different time frames after admission. For secondary end points, we considered the absolute BP indices on admission, absolute BP indices at different time points as well as BPV indices within the different time frames on good outcome at discharge, mortality at discharge, and mortality at follow-up after 3 months.

### Statistical Analysis

Data were collected and evaluated using Excel (Microsoft, Redmond, WA) spreadsheet software. Statistical analysis was performed using SPSS (IBM, Armonk, NY). An exploratory analysis was conducted on baseline characteristics, periprocedural time, and outcome parameters. The Shapiro–Wilk test was used for the assessment of data normality. Pearson’s χ^2^ test was used to examine differences between groups characteristics for categorical variables, whereas the Mann–Whitney *U*-test was used for the continuous ones. Values are presented as medians with interquartile ranges (IQRs) for continuous variables and as the total number with the percentage for categorical variables. BPV was calculated from continuous data using R (current version, R 4.2.0). After data preparation and import of continuous BP data, data quality was ensured by cleaning and preprocessing, including handling missing values and outliers, and the continuous BP readings were transformed into a format suitable for analysis. Common measures included the SD and CV.

We performed univariate regression analyses regarding different parameters, including absolute BP and BPV indices and other risk factors, for good outcome at 3 months’ follow-up. To evaluate the independent association between BPV indices and good functional outcomes, we conducted a logistic regression using three distinct models to understand the impact of adding different BPV indices during different time periods on the outcomes. Model 1, referred to as the baseline model, incorporated demographics and baseline characteristics to establish a foundational understanding of the patient population. This model served as a comparator for assessing additional variables’ impacts on the predictive accuracy or outcome of interest. Model 2 expanded on the baseline model by incorporating each BPV index measured during different time periods. This addition aimed to evaluate whether fluctuations in BP, beyond the baseline demographic and characteristic data, provide significant predictive value or insights into patient outcomes. The comparison between model 1 and model 2 allowed for a comprehensive analysis of how BPV indices contribute to the model’s overall efficacy and the potential implications for clinical practice. Model 3, termed the full model, was developed in response to findings that individual BPV indices did not demonstrate significant associations with good outcome in ICH. Therefore, this model incorporated both systolic and diastolic BPV (SD) during the critical initial 0–24 h post ICH onset, following a thorough evaluation of the potential implications. By integrating comprehensive BPV measures, model 3 sought to offer a more nuanced understanding of BPV’s role in patient outcomes, especially considering the acute phase after ICH. The progression from model 1 to model 3 reflected an iterative approach to model development, starting from basic demographic information to a more complex integration of BPV indices. This strategy allowed for a detailed examination of how variations in BP, particularly in the immediate aftermath of ICH, might influence patient trajectories and outcomes. The level of statistical significance was set to *p* < 0.05.

### Ethics Statement

This study was approved by the local ethics committee (protocol 545/2022BO2) and was performed in accordance with the ethical standards laid down in the 1964 Declaration of Helsinki and its later amendments. Participant individual informed consent has been waived in terms of the clinic-wide consent regarding the use of deidentified routine treatment data for research purposes.

## Results

### Patients Characteristics

Our study included a cohort of 261 patients with ICH (mean age 69.6 ± 16.5 years, 39.1% female, median National Institutes of Health Stroke Scale [NIHSS] score on admission 6 [IQR 2–12], median ICH score 1 [IQR 0–2]) and data on functional outcome at follow-up after 3 months. A large proportion of the patients had arterial hypertension (80.8%), and other prevalent risk factors included diabetes mellitus (18.8%) and atrial fibrillation (25.7%). Upon admission, the median ICH volume was 17.4 ± 24.6 mL, and a notable percentage of patients were under anticoagulant therapy (24.9%), reflecting the complex medical profiles typically associated with ICH (for further details, see Table 2).

**Table 2:** Descriptive comparison of baseline characteristics, interventions, and functional outcome of patients with ICH, based on good functional outcome (mRS 0–2) at 90 days’ follow-up

	All patients with ICH *N* = 261	Patients with ICH with good outcome *n* = 106	Patients with ICH with poor outcome (mRS ≥ 3) *n* = 155	*p* value
Age, y mean ± SD	69.6 ± 15.2	63.2 ± 16.5	74.0 ± 12.5	< 0.001
Sex, male, n (%)	159 (52.1%)	59 (55.7%)	75 (48.4%)	0.250
pmRS, median (IQR)	0 (0, 2)	0 (0, 0)	1 (0, 3)	< 0.001
Baseline clinical variables/scales on admission				
NIHSS median (IQR)	6 (2, 12)	3 (1, 6)	9 (5, 15)	< 0.001
ICH score median (IQR)	1 (0, 2)	1 (0, 1)	2 (1, 2)	< 0.001
Risk factors n (%)				
Arterial hypertension	211 (80.8%)	80 (75.5%)	131 (84.5%)	0.034
Diabetes mellitus	49 (18.8%)	7 (6.6%)	42 (27.1%)	< 0.001
Atrial fibrillation	67 (25.7%)	17 (16.0%)	52 (33.5%)	< 0.001
Hypercholesterolemia	51 (19.5%)	15 (14.2%)	34 (21.9%)	0.240
Smoking	37 (14.2%)	17 (16.0%)	20 (12.9%)	0.478
Alcohol	21 (8.0%)	8 (7.5%)	13 (8.4%)	0.807
Obesity (BMI > 30)	56 (21.6%)	15 (14.2%)	41 (26.5%)	0.017
Coronary heart disease	42 (16.1%)	7 (6.6%)	35 (22.6%)	< 0.001
Chronic renal failure	30 (11.5%)	7 (6.6%)	23 (14.8%)	0.041
CHA2DS2-Vasc Score	3 (2, 4)	2 (1, 4)	4 (2, 5)	< 0.001
Anticoagulation on admission, n (%)	65 (24.9%)	16 (15.1%)	49 (31.6%)	0.002
Antiplatelet therapy on admission, n (%)	60 (2.0%)	16 (15.1%)	44 (28.4%)	0.012
Laboratory findings on admission				
INR mean ± SD	1.1 ± 0.4	1.1 ± 0.2	1.2 ± 0.4	0.084
PTT mean ± SD	24.6 ± 5.3	24.4 ± 4.8	24.8 ± 5.6	0.470
Thrombocytes mean ± SD	216 ± 79	220 ± 79	214 ± 80	0.497
Imaging, n (%)				
Supratentorial localization				
Deep	127 (48.7%)	43 (40.6%)	84 (54.2%)	0.031
Lobar	101 (38.7%)	44 (41.5%)	57 (36.8%)	0.442
Infratentorial localization				
Brainstem	16 (6.1%)	7 (6.6%)	9 (5.8%)	0.793
Cerebellum	28 (10.7%)	14 (13.2%)	14 (9.0%)	0.286
Siderosis	21 (8.0%)	8 (7.5%)	13 (8.4%)	0.807
FAZEKAS PWM median (IQR)	1 (0, 1)	1 (0, 1)	1 (0, 2)	0.131
FAZEKAS DWM median (IQR)	1 (0, 2)	1 (0, 2)	1 (0, 2)	0.327
ICH Volume [cm^3^/ml] mean ± SD on admission	17.4 ± 24.6	10.6 ± 17.6	22.0 ± 4.3	< 0.001
IVH on admission	1 (0.4%)	0 (0%)	1 (0.6%)	0.409
IVH secondary	92 (35.2%)	22 (20.8%)	70 (45.2%)	< 0.001
Early hematoma expansion	37 (14.2%)	11 (10.4%)	26 (16.8%)	0.147
Etiology CLAS-ICH subtypes, n (%)				
Well defined cause (grade 1)	110 (42.1%)	50 (47.2%)	60 (38.7%)	0.004
A1	56 (21.5%)	23 (21.7%)	33 (21.3%)	
C1	18 (6.9%)	3 (2.8%)	15 (9.7%)	
S1	35 (13.4%)	24 (22.6%)	11 (7.1%)	
Possible cause (grade 2)	74 (28.4%)	34 (32.1%)	40 (25.8%)	
A2	93 (35.6%)	37 (34.9%)	56 (36.1%)	
C2	11 (4.2%)	6 (5.7%)	5 (3.2%)	
M2	19 (7.3%)	9 (8.5%)	10 (6.5%)	
S2	6 (2.3%)	3 (2.8%)	3 (1.9%)	
Multiple causes (≥ 2 grades 1 or 2)	7 (2.7%)	4 (3.8%)	4 (1.9%)	
No complete work-up (grade 9)	70 (26.8%)	18 (17.0%)	52 (33.5%)	
Interventions n (%)				
Any acute intervention using medication for coagulation/ clotting	73 (28.0%)	13 (12.3%)	60 (38.7%)	< 0.001
TK	15 (5.7%)	4 (3.8%)	11 (7.1%)	0.259
Minirin	15 (5.7%)	2 (1.9%)	13 (8.4%)	0.027
PPSB	40 (15.3%)	6 (5.7%)	34 (21.9%)	< 0.001
Andexanet alpha	12 (4.6%)	1 (0.9%)	11 (7.1%)	0.020
Idarucizumab	1 (0.4%)	1 (0.9%)	0 (0%)	0.227
Vit K	7 (2.7%)	2 (1.9%)	5 (3.2%)	0.513
Protamin	3 (1.1%)	2 (1.9%)	1 (0.6%)	0.357
ICU-stay days (mean ± SD)	9.9 ± 9.3	6.6 ± 5.5	12.1 ± 10.6	< 0.001
Hospital stay days (mean ± SD)	14.6 ± 10.4	12.8 ± 10.1	15.9 ± 10.3	0.015
Neurosurgical procedure n (%)	32 (12.3%)	13 (12.3%)	19 (12.3%)	0.999
EVD n (%)	36 (13.8%)	10 (9.4%)	26 (16.8%)	0.092
Infection, n (%)	137 (52.5%)	32 (30.2%)	105 (67.7%)	< 0.001
Intubation, n (%)	96 (36.8%)	34 (32.1%)	62 (40.0%)	0.140
Presence of delirium	103 (39.5%)	24 (22.6%)	79 (51.0%)	< 0.001
NIHSS follow-up (24 h)	6 (2, 13)	3 (1, 5)	10 (5, 15)	< 0.001
Discharge				
NIHSS median (IQR)	5 (1, 11)	2 (0, 4)	9 (4, 14)	< 0.001
mRS median (IQR)	4 (3, 5)	2 (1, 4)	5 (4, 5)	< 0.001
Mortality	9 (3.4%)	0 (0%)	9 (5.8%)	0.011
Good outcome	57 (21.8%)	56 (52.8%)	1 (0.6%)	< 0.001
Poor outcome	204 (78.2%)	50 (47.2%)	154 (99.4%)	
Follow-Up at 90 days				
mRS median (IQR)	3 (1, 5)	1 (0, 2)	4 (4, 5)	< 0.001
Mortality	32 (12.3%)	0 (0%)	32 (20.6%)	< 0.001
Good outcome	106 (40.6%)	106 (100%)	0 (0%)	
Poor outcome	155 (59.4%)	0 (0%)	155 (100%)	

Statistically significant values are highlighted

BMI, body mass index; DWM, deep white matter; EVD, external ventricular drain; GCS; glascow coma scale; ICH, intracerebral hemorrhage; ICU, intensive care unit; International Nomalized Ratio; IVH, intraventricular hemorrhage; NIHSS, national institutes of health stroke scale; OAC, oral anticoagulants; PTT, Partial Thromboplastin Time; pmRS, modified Rankin scale before the index event; mRS, modified Rankin scale; SD, standard deviation; TK, thrombocyte concentrate; INR

^Mann–Whitney U-test for nonnormally or ordinal variables

^*^Chi-square test for normally distributed and continuous variables

^*^Percent values are represented as decimal numbers. For example, 0.25 means that 25% of the values are above the specified threshold

### Primary End Point/Good Functional Outcome at 90 Days’ Follow-up

Patients with ICH with good outcomes (*n* = 106) at follow-up were younger than those with poor outcomes (*n* = 155) (63.2 ± 16. vs. 74.0 ± 12.5, *p* < 0.001). The good outcome group also had significantly lower mRS scores before the index event and better baseline clinical variables, such as a lower median NIHSS score (3 vs. 9), a higher Glasgow Coma Scale score (15 vs. 14), and a lower ICH score (1 vs. 2, all *p* < 0.001). Cardiovascular risk factors, such as DM, atrial fibrillation, obesity (body mass index > 30), and coronary heart disease, as well as chronic renal failure were less prevalent in the good outcome group. Further, patients with poor outcome were more likely to have been on anticoagulation therapy upon admission (15.1% vs. 31.6%, *p* = 0.002). Consequently, patients with good outcome had smaller ICH volumes on admission (10.6 ± 17.6 vs. 22.0 ± 4.3, *p* < 0.001) and a lower incidence of intraventricular hemorrhage, both on admission and secondary during the clinical course (each *p* < 0.001). However, early hematoma expansion was not significantly different between the groups (*p* = 0.147). In terms of interventions, patients with poor outcomes had more frequent acute interventions using medication for coagulation/clotting (*p* < 0.001) and had longer stays in the ICU and the hospital (both *p* < 0.015).

Overall, mortality rates were significantly higher for patients with poor outcomes at both discharge and follow-up (*p* = 0.011 and *p* < 0.001, respectively), whereas good functional outcome was more likely in the other group (*p* < 0.001) (for further details, see Table 2). All patients who died within the 90-day follow-up period passed away because of withdrawal of life support. Except for one patient, the patients died at discharge or were transferred with palliative intent. In addition, the mRS score distribution between discharge and 90-day follow-up is shown in Fig. 3 in the supplement.

### First BP on Admission and Absolute BP Indices Throughout the First 72 Hours Post Admission

Patients with ICH with a good outcome had significantly lower systolic BP on admission (mean 150.9 ± 23.7 vs. 162.1 ± 29.7 mm Hg, *p* = 0.002), whereas diastolic BP was numerically but not significantly higher (mean 86.4 ± 19.4 vs. 84.7 ± 20.5 mm Hg, *p* = 0.525). As time progressed, the difference with lower systolic BP and higher diastolic BP became more pronounced (for further details, see Table 3), whereas there were no differences in MAP. Within the first 2 h, systolic BP mean values were significantly lower for patients with good outcomes (mean 147.1 ± 20.8 vs. 154.5 ± 23.1 mm Hg, *p* = 0.009), and this pattern persisted, with significant differences observed in every subsequent time frame up to 72 h. Furthermore, the analysis of the absolute proportion of systolic BP values exceeding 140 mm Hg revealed that patients with good outcomes consistently showed lower proportions across all time frames, particularly notable during the initial 72 h (0.47 ± 0.27 vs. 0.57 ± 0.20, *p* < 0.001). Diastolic BP values also reflected significant differences, particularly in the earlier periods (0–2 h, 0–8 h, and 0–12 h), when patients with good outcomes had higher mean diastolic BP, indicating a potential trend toward better outcomes with a slightly elevated diastolic pressure during acute management phases. For instance, during the 0–12-h time frame, diastolic BP was significantly higher for those with good outcomes (mean 74.6 ± 13.0 vs. 70.1 ± 10.7 mm Hg, *p* = 0.003).

**Table 3:** Descriptive comparison of absolute blood pressure in patients with ICH, based on good functional outcome (mRS 0–2) at 90 days’ follow-up

	All patients with ICH *N* = 305	Patients with ICH with good outcome *n* = 106	Patients with ICH with poor outcome *n* = 155	*p* value
On admission				
Systolic BP mean ± SD (mmHg)	157.5 ± 27.9	150.9 ± 23.7	162.1 ± 29.7	0.002
diastolic BP mean ± SD (mmHg)	85.4 ± 20.0	86.4 ± 19.4	84.7 ± 20.5	0.525
MAP mean ± SD (mmHg)	111.2 ± 23.2	110.2 ± 17.3	111.9 ± 24.6	0.560
Time frame 0–2 h				
Systolic BP mean ± SD (mmHg)	151.5 ± 22.5	147.1 ± 20.8	154.5 ± 23.1	0.009
Proportion of values exceeding 140 mmHg systolic (%)*	0.68 ± 0.35	0.65 ± 0.39	0.69 ± 0.32	0.407
Diastolic BP mean ± SD (mmHg)	78.6 ± 15.2	80.9 ± 14.0	77.1 ± 15.8	0.047
MAP mean ± SD (mmHg)	105.9 ± 17.6	106.8 ± 16.3	105.4 ± 18.4	0.551
Time frame 0–8 h				
Systolic BP mean ± SD (mmHg)	145.6 ± 17.2	142.3 ± 18.6	147.8 ± 15.9	0.012
Proportion of values exceeding 140 mmHg systolic (%)*	0.58 ± 0.30	0.55 ± 0.34	0.60 ± 0.27	0.130
Diastolic BP mean ± SD (mmHg)	73.5 ± 12.3	76.0 ± 13.1	71.8 ± 11.4	0.008
MAP mean ± SD (mmHg)	100.3 ± 13.3	101.7 ± 14.5	99.4 ± 12.4	0.161
Time frame 0–12 h				
Systolic BP mean ± SD (mmHg)	143.5 ± 15.7	140.9 ± 17.3	145.3 ± 14.3	0.025
Proportion of values exceeding 140 mmHg systolic (%)*	0.55 ± 0.29	0.52 ± 0.33	0.57 ± 0.25	0.231
Diastolic BP mean ± SD (mmHg)	71.9 ± 11.9	74.6 ± 13.0	70.1 ± 10.7	0.003
MAP mean ± SD (mmHg)	98.5 ± 12.4	100.1 ± 13.8	97.4 ± 11.2	0.003
Time frame 0–24 h				
Systolic BP mean ± SD (mmHg)	141.4 ± 13.9	138.5 ± 15.6	143.4 ± 12.4	0.006
Proportion of values exceeding 140 mmHg systolic (%)*	0.52 ± 0.26	0.48 ± 0.30	0.54 ± 0.23	0.080
Diastolic BP mean ± SD (mmHg)	70.0 ± 10.9	72.5 ± 11.8	68.410.0	0.002
MAP mean ± SD (mmHg)	96.4 ± 11.0	97.7 ± 12.3	95.5 ± 10.0	0.119
Time frame 0–48 h				0.095
Systolic BP mean ± SD (mmHg)	140.9 ± 13.0	137.8 ± 14.5	143.0 ± 11.4	0.001
Proportion of values exceeding 140 mmHg systolic (%)*	0.51 ± 0.24	0.46 ± 0.28	0.55 ± 0.20	0.006
Diastolic BP mean ± SD (mmHg)	69.1 ± 10.2	71.7 ± 11.2	67.3 ± 9.1	< 0.001
MAP mean ± SD (mmHg)	95.7 ± 9.9	96.9 ± 11.2	94.8 ± 8.8	0.105
Time frame 0–72 h				
Systolic BP mean ± SD (mmHg)	141.2 ± 12.7	138.0 ± 14.2	143.4 ± 11.0	< 0.001
Proportion of values exceeding 140 mmHg systolic (%)*	0.52 ± 0.23	0.46 ± 0.27	0.56 ± 0.19	0.614
Diastolic BP mean ± SD (mmHg)	69.1 ± 9.9	71.9 ± 10.4	67.3 ± 9.1	< 0.001
MAP mean ± SD (mmHg)	95.7 ± 9.4	96.9 ± 10.4	94.9 ± 8.5	0.090

Statistically significant values are highlighted

BP, blood pressure; HR, heart rate; MAP, middle arterial pressure; SD, standard deviation

^Mann–Whitney U-test for nonnormally or ordinal variables

^*^Chi-square test for normally distributed and continuous variables

^*^Percent values are represented as decimal numbers. For example, 0.25 means that 25% of the values are above the specified threshold

### Relationship Between Patient-Related Risk Factors, Acute ICH Treatment, and Good Functional Outcome at Follow-up

Key findings from the univariate analysis examining factors associated with a good functional outcome after ICH were as follows: age (odds ratio [OR] 0.950, 95% confidence interval [CI] 0.932–0.968, *p* < 0.001) and cardiovascular risk factors as indicated by CHA2DS2VAsc Score (OR 0.638, 95% CI 0.540–0.755, *p* < 0.001) were inversely associated with good outcome. Higher NIHSS score (OR 0.801, 95% CI 0.750–0.855, *p* < 0.001), higher ICH score (OR 0.419, 95% CI 0.311–0.563, *p* < 0.001), and higher ICH volume on admission (OR 0.972, 95% CI 0.957–0.988, *p* < 0.001) were significantly linked to worse outcomes. Early HE showed a nonsignificant trend to reduce the odds of good functional outcome. Any acute intervention using antagonization for anticoagulation showed a strong positive association with a good outcome (OR 0.221, 95% CI 0.114–0.430, *p* < 0.001), whereas complications during hospital stay reduced the likelihood for good outcome (for further results, see Table 4).

**Table 4:** Univariate logistic regression analysis for association of baseline characteristics, interventions, BP parameters, and good clinical outcome at follow-up

	OR	Lower confidence interval limit	Upper confidence interval limit	*p* value
Age	0.950	0.932	0.968	< 0.001
Sex	0.747	0.455	1.226	0.249
pmRS	0.375	0.265	0.531	< 0.001
Risk factors				
Arterial hypertension	0.564	0.303	1.049	0.070
Diabetes mellitus	0.190	0.082	0.443	< 0.001
HCL	0.680	0.357	1.293	0.240
Atrial fibrillation	0.327	0.172	0.619	< 0.001
CAD	0.242	0.103	0.569	< 0.001
Obesity (BMI > 30)	1.284	0.545	3.026	0.568
Smoking	1.289	0.640	2.596	0.477
CHA2DS2VAcScore	0.638	0.540	0.755	< 0.001
Baseline clinical variables/scales				
NIHSS on admission	0.801	0.750	0.855	< 0.001
GCS	1.223	1.106	1.352	< 0.001
ICH score	0.419	0.311	0.563	< 0.001
HR on admission	0.993	0.977	1.008	0.359
Systolic BP on admission	0.985	0.975	0.995	0.002
Diastolic BP on admission	1.004	0.991	1.017	0.524
Location at baseline				
Deep localization	0.577	0.350	0.951	0.031
Lobar localization	1.220	0.736	2.023	0.441
Brainstem	1.147	0.414	3.181	0.792
Cerebellum	1.533	0.698	3.363	0.287
IVH on admission	NA	NA	NA	NA
ICH Volume on admission	0.972	0.957	0.988	< 0.001
Early hematoma expansion	0.574	0.271	1.220	0.149
Secondary IVH	0.314	0.178	0.554	< 0.001
Patients under oral anticoagulants	0.385	0.205	0.722	0.003
Patients under antiplatelet therapy	0.448	0.237	0.847	0.013
Interventions				
Any acute intervention using medication for coagulation/ clotting	0.221	0.114	0.430	< 0.001
Neurosurgical procedure	1.001	0.471	2.125	0.999
EVD	0.517	0.238	1.123	0.095
Infection during hospital stay	0.206	0.121	0.351	< 0.001
Presence of delirium	0.282	0.162	0.490	< 0.001
Absolute BP indices				
Systolic BP mean (0–2 h)	0.985	0.973	0.997	0.012
Diastolic BP mean (0–2 h)	1.017	1.000	1.034	0.055
Systolic BP mean (0–8 h)	0.981	0.967	0.996	0.013
Diastolic BP mean (0–8 h)	1.029	1.008	1.051	0.007
Systolic BP mean (0–12 h)	1.0982	0.966	0.998	0.027
Diastolic BP mean (0–12 h)	1.003	1.010	1.056	0.004
Systolic BP mean (0–24 h)	0.975	0.957	0.993	0.007
Diastolic BP mean (0–24 h)	1.036	1.012	1.061	0.003
Systolic BP mean (0–48 h)	0.968	0.949	0.988	0.002
Diastolic BP mean (0–48 h)	1.044	1.018	1.071	< 0.001
Proportion of values exceeding 140 mmHg systolic (%)	0.228	0.079	0.660	0.006
Systolic BP mean (0–72 h)	0.965	0.945	0.986	0.001
Diastolic BP mean (0–72 h)	1.050	1.022	1.079	< 0.001
Blood pressure variability indices				
Systolic BP SD (0–2 h)	0.975	0.953	0.997	0.026
Systolic BP SD (0–8 h)	0.955	0.928	0.981	0.001
Systolic BP CV (0–8 h)	0.004	0.000	0.235	0.008
Diastolic BP CV (0–8 h)	0.022	0.001	0.446	0.013
Systolic BP SD (0–12 h)	0.946	0.918	0.976	< 0.001
Systolic BP CV (0–12 h)	0.001	0.000	0.073	0.002
Diastolic BP SD (0–12 h)	0.961	0.922	1.001	0.054
Diastolic BP CV (0–12 h)	0.012	0.000	0.299	0.007
Systolic BP SV (0–24 h)	0.973	0.944	1.002	0.065
Systolic BP SD (0–24 h)	0.938	0.905	0.972	< 0.001
Systolic BP CV (0–24 h)	0.00001	0.0000001	0.044	0.002
Diastolic BP SV (0–24 h)	0.985	0.945	1.026	0.466
Diastolic BP CV (0–24 h)	0.020	0.001	0.637	0.027
Systolic BP SV (0–48 h)	0.971	0.937	1.006	0.099
Systolic BP SD (0–48 h)	0.916	0.875	0.959	< 0.001
Systolic BP CV (0–48 h)	0.00001	0.0000001	0.010	< 0.001
Diastolic BP SV (0–48 h)	1.005	0.958	1.054	0.847
Systolic BP SV (0–72 h)	0.968	0.930	1.006	0.101
Systolic BP SD (0–72 h)	0.915	0.871	0.960	< 0.001
Systolic BP CV (0–72 h)	0.00001	0.0000001	0.018	0.002
Diastolic BP SV (0–72 h)	1.013	0.963	1.066	0.613

AF, atrial fibrillation; BP, blood pressure; BMI, body mass index; CAD, coronary artery disease; EVD, external ventricular drain; GCS, Glasgow Coma Scale; HCL, Hypercholesterolemia; HR, heart rate; ICH, intracerebral hemorrhage; IVH, intraventricular hemorrhage; NA, nonapplicable; NIHSS, National Institutes of Health Stroke Scale; OAC, oral anticoagulants; pmRS, modified Rankin Scale before the index event; mRS, modified Rankin Scale

### Relationship Between Absolute BP and Good Functional Outcome at Follow-up

A significant association was observed between lower average systolic BP and better outcomes in the first 0–2, 0–8, and 0–24 h, with ORs of 0.985, 0.981, and 0.975, respectively, suggesting that lower systolic BP values in these early time windows are associated with a higher likelihood of a good outcome (*p* = 0.012, 0.013, and 0.007, respectively). Higher average diastolic BP values in the first 0–8, 0–2, and 0–48 h showed a significant association with better outcomes (ORs of 1.029, 1.036, and 1.044; *p* = 0.007, *p* = 0.003, and *p* < 0.001). Further, a lower proportion of systolic BP values exceeding 140 mm Hg showed a significant association (OR 0.228, *p* = 0.006) with a good outcome. In the long-term observation up to 48 and 72 h after admission, results continued to indicate that both lower systolic and higher diastolic average values were associated with better outcomes (ORs for systolic BP: 0.968 and 0.965; ORs for diastolic BP: 1.044 and 1.050) (for further results, see Table 5).

**Table 5:** Descriptive comparison of blood pressure variability indices in patients with ICH, based on good functional outcome (mRS 0–2) at 90 days’ follow-up

	All patients with ICH *n* = 305	Patients with ICH with good outcome *n* = 106	Patients with ICH with poor outcome *n* = 155	*p* value
Time frame 0–2 h				
Systolic BP SV mean ± SD (mmHg)	17.8 ± 17.8	15.5 ± 17.6	19.3 ± 17.9	0.101
Systolic BP SD mean ± SD (mmHg)	16.6 ± 13.9	14.2 ± 13.9	18.3 ± 13.6	0.021
Systolic BP CV, Mean ± SD*	0.11 ± 0.11	0.10 ± 0.12	0.12 ± 0.11	0.122
Diastolic BP SV mean ± SD (mmHg)	11.9 ± 11.5	11.3 ± 11.2	12.4 ± 11.7	0.447
Diastolic BP SD mean ± SD (mmHg)	11.1 ± 8.7	10.2 ± 8.3	11.6 ± 8.9	0.191
Diastolic BP CV, Mean ± SD*	0.14 ± 0.12	0.13 ± 0.11	0.15 ± 0.12	0.103
MAP SV mean ± SD (mmHg)	14.7 ± 15.1	13.5 ± 15.5	15.5 ± 14.9	0.330
MAP SD mean ± SD (mmHg)	13.7 ± 11.8	12.4 ± 12.1	14.6 ± 11.6	0.151
MAP CV, Mean ± SD*	0.14 ± 0.12	0.12 ± 0.12	0.14 ± 0.12	0.144
Time frame 0–8 h				
Systolic BP SV mean ± SD (mmHg)	17.1 ± 12.9	15.4 ± 12.8	18.3 ± 12.8	0.074
Systolic BP SD mean ± SD (mmHg)	18.9 ± 11.3	16.0 ± 11.3	21.3 ± 9.8	< 0.001
Systolic BP CV, Mean ± SD*	0.13 ± 0.08	0.11 ± 0.08	0.15 ± 0.07	0.007
Diastolic BP SV mean ± SD (mmHg)	11.1 ± 8.3	10.5 ± 7.9	11.4 ± 8.5	0.409
Diastolic BP SD mean ± SD (mmHg)	12.2 ± 7.2	11.2 ± 6.7	12.9 ± 7.5	0.053
diastolic BP CV, Mean ± SD*	0.17 ± 0.09	0.14 ± 0.09	0.18 ± 0.10	0.011
MAP SV mean ± SD (mmHg)	14.2 ± 11.7	13.3 ± 12.4	14.8 ± 11.3	0.323
MAP SD mean ± SD (mmHg)	15.7 ± 10.3	14.1 ± 10.4	16.7 ± 10.2	0.041
MAP CV, Mean ± SD*	0.16 ± 0.10	0.14 ± 0.10	0.17 ± 0.10	0.024
Time frame 0–12 h				
Systolic BP SV mean ± SD (mmHg)	16.8 ± 11.5	15.3 ± 11.3	17.8 ± 11.5	0.081
Systolic BP SD mean ± SD (mmHg)	19.3 ± 10.2	16.5 ± 10.2	21.3 ± 8.8	0.040
Systolic BP CV, Mean ± SD*	0.13 ± 0.07	0.12 ± 0.07	0.15 ± 0.07	0.001
Diastolic BP SV mean ± SD (mmHg)	16.7 ± 7.4	10.1 ± 7.2	11.0 ± 7.6	0.337
Diastolic BP SD mean ± SD (mmHg)	12.1 ± 6.6	11.2 ± 6.2	12.8 ± 6.8	< 0.001
diastolic BP CV, Mean ± SD*	0.17 ± 0.09	0.15 ± 0.08	0.18 ± 0.09	0.005
MAP SV mean ± SD (mmHg)	13.6 ± 10.3	12.9 ± 10.9	14.1 ± 9.9	0.360
MAP SD mean ± SD (mmHg)	15.6 ± 9.4	14.2 ± 9.5	16.6 ± 9.2	0.050
MAP CV, Mean ± SD*	0.16 ± 0.09	0.14 ± 0.10	0.17 ± 0.09	0.026
Time frame 0–24 h				
Systolic BP SV mean ± SD (mmHg)	16.4 ± 9.4	15.1 ± 9.1	17.4 ± 9.6	0.029
Systolic BP SD mean ± SD (mmHg)	19.3 ± 8.3	17.1 ± 8.6	20.9 ± 7.8	< 0.001
Systolic BP CV, Mean ± SD*	0.14 ± 0.06	0.12 ± 0.06	0.15 ± 0.05	0.002
Diastolic BP SV mean ± SD (mmHg)	10.3 ± 6.2	10.0 ± 5.9	10.5 ± 6.3	0.467
Diastolic BP SD mean ± SD (mmHg)	11.7 ± 5.6	11.4 ± 5.5	12.3 ± 5.7	0.218
diastolic BP CV, Mean ± SD*	0.17 ± 0.08	0.16 ± 0.07	0.18 ± 0.08	0.022
MAP SV mean ± SD (mmHg)	13.3 ± 8.3	12.7 ± 8.7	13.8 ± 8.1	0.324
MAP SD mean ± SD (mmHg)	15.6 ± 7.5	14.5 ± 7.8	16.2 ± 7.2	0.067
MAP CV, Mean ± SD*	0.16 ± 0.08	0.15 ± 0.08	0.17 ± 0.07	0.029
Time frame 0–48 h				0.095
Systolic BP SV mean ± SD (mmHg)	16.2 ± 7.7	15.3 ± 7.1	16.9 ± 8.0	0.095
Systolic BP SD mean ± SD (mmHg)	19.0 ± 6.6	17.1 ± 6.9	20.4 ± 6.1	< 0.001
Systolic BP CV, Mean ± SD*	0.13 ± 0.04	0.12 ± 0.05	0.14 ± 0.04	< 0.001
Diastolic BP SV mean ± SD (mmHg)	10.4 ± 5.2	10.5 ± 5.2	10.4 ± 5.3	0.848
Diastolic BP SD mean ± SD (mmHg)	11.8 ± 4.7	11.6 ± 4.7	11.9 ± 4.6	0.586
diastolic BP CV, Mean ± SD*	0.17 ± 0.07	0.16 ± 0.07	0.18 ± 0.06	0.099
MAP SV mean ± SD (mmHg)	13.3 ± 6.8	12.9 ± 7.0	13.5 ± 6.6	0.471
MAP SD mean ± SD (mmHg)	11.8 ± 4.7	14.3 ± 6.3	15.8 ± 5.7	< 0.001
MAP CV, Mean ± SD*	0.16 ± 0.06	0.15 ± 0.07	0.17 ± 0.06	0.024
Time frame 0–72 h				
Systolic BP SV mean ± SD (mmHg)	16.1 ± 6.9	15.2 ± 6.2	16.7 ± 7.3	0.043
Systolic BP SD mean ± SD (mmHg)	18.9 ± 6.0	17.3 ± 6.2	20.1 ± 5.6	< 0.001
Systolic BP CV, Mean ± SD*	0.13 ± 0.04	0.12 ± 0.05	0.14 ± 0.04	0.002
Diastolic BP SV mean ± SD (mmHg)	10.6 ± 4.9	10.8 ± 4.7	10.5 ± 5.0	0.614
Diastolic BP SD mean ± SD (mmHg)	11.8 ± 4.4	11.7 ± 4.4	11.9 ± 4.4	0.069
diastolic BP CV, Mean ± SD*	0.17 ± 0.06	0.16 ± 0.06	0.18 ± 0.06	0.094
MAP SV mean ± SD (mmHg)	13.1 ± 5.9	12.9 ± 5.9	13.3 ± 5.9	0.528
MAP SD mean ± SD (mmHg)	15.0 ± 5.4	14.3 ± 5.5	15.5 ± 5.2	< 0.001
MAP CV, Mean ± SD*	0.16 ± 0.05	0.15 ± 0.06	0.16 ± 0.05	0.022
Time frame 8–16 h				
Systolic BP SV mean ± SD (mmHg)	14.3 ± 8.4	12.5 ± 7.1	15.5 ± 9.0	0.005
Systolic BP SD mean ± SD (mmHg)	14.1 ± 6.9	12.0 ± 6.2	15.6 ± 7.0	< 0.001
Systolic BP CV, Mean ± SD*	0.10 ± 0.05	0.09 ± 0.04	0.11 ± 0.05	< 0.001
Diastolic BP SV mean ± SD (mmHg)	8.2 ± 5.5	7.3 ± 3.7	8.7 ± 6.3	0.036
Diastolic BP SD mean ± SD (mmHg)	7.7 ± 4.4	6.9 ± 2.3	8.3 ± 5.2	0.011
diastolic BP CV, Mean ± SD*	0.12 ± 0.07	0.10 ± 0.04	0.13 ± 0.08	0.002
MAP SV mean ± SD (mmHg)	10.8 ± 7.7	9.5 ± 5.9	11.7 ± 8.6	0.029
MAP SD mean ± SD (mmHg)	10.6 ± 6.5	9.3 ± 5.2	11.4 ± 7.1	0.011
MAP CV, Mean ± SD*	0.11 ± 0.07	0.10 ± 0.05	0.13 ± 0.07	0.003

Statistically significant values are highlighted

BP, blood pressure; CV, coefficient of variation; MAP, middle arterial pressure; SD, standard deviation; SV, successive variability

^Mann–Whitney U-test for nonnormally or ordinal variables

^*^Chi-square test for normally distributed and continuous variables

^*^Percent values are represented as decimal numbers. For example, 0.25 means that 25% of the values are above the specified threshold

### BPV

BPV was different regarding patients with ICH with good outcome vs. poor outcome throughout the first 72 h after admission. In detailed analysis of the first 24 h after admission, there was a clear trend of lower systolic and diastolic BPV without reaching statistical significance during the first 2 h after admission, whereas in the time periods 0–8 h, 0–12 h, and especially 8–16 h, the differences were significantly different. Across the initial 2-h period, systolic BP SV showed a numerical but not significant difference (15.5 ± 17.6 vs. 19.3 ± 17.9 mm Hg, *p* = 0.101); however, SD of systolic BP was significantly different (14.2 ± 13.9 vs. 18.3 ± 13.6 mm Hg, *p* = 0.021). In the time period 0–8 h, this trend persisted, now also showing significant differences in diastolic BPV regarding CV (0.17 ± 0.09 vs. 0.18 ± 0.10 mm Hg, *p* = 0.011) and SD (14.1 ± 10.4 vs. 16.7 ± 10.2 mm Hg, *p* = 0.041). This trend persisted across most metrics, with systolic and diastolic BPV indicators similarly reflecting differences between outcome groups at various time intervals (for further details, see Table 3).

### Relationship Between BPV Indices and Good Functional Outcome at Follow-up

The univariate analysis for good outcome in ICH with different BPV indices showed a consistent pattern: lower variability in BP, particularly in systolic BP, was associated with better outcomes. Lower SD values at various time intervals (0–2 h, 0–8 h, 0–12 h, 0–24 h, 0–48 h, and 0–72 h) were significantly associated with a good outcome, indicating that less fluctuation in systolic BP is beneficial. The statistical significance of these associations strengthened over longer periods, especially notable at 0–8 h (OR 0.955, *p* = 0.001) and beyond. Extremely low CV values (indicative of very little relative variability) across different time intervals (0–8 h, 0–12 h, 0–24 h, 0–48 h, and 0–72 h) were strongly associated with good outcomes, with ORs close to zero and highly significant *p* values, suggesting that relative stability in systolic BP is crucial for recovery. Similar to systolic BP, lower diastolic BP CV at 0–8 h and 0–12 h was associated with better outcomes, although the association was less consistent across all time frames. There was a nonsignificant trend toward lower SV at 0–24 h and 0–72 h being associated with better outcomes, indicating that less immediate fluctuation in systolic BP might be beneficial, although the evidence was less robust compared to SD and CV measures.

### Multivariate Analysis of Absolute BP and BPV Indices and Their Independent Association with Good Functional Outcomes

**Fig. 1:**
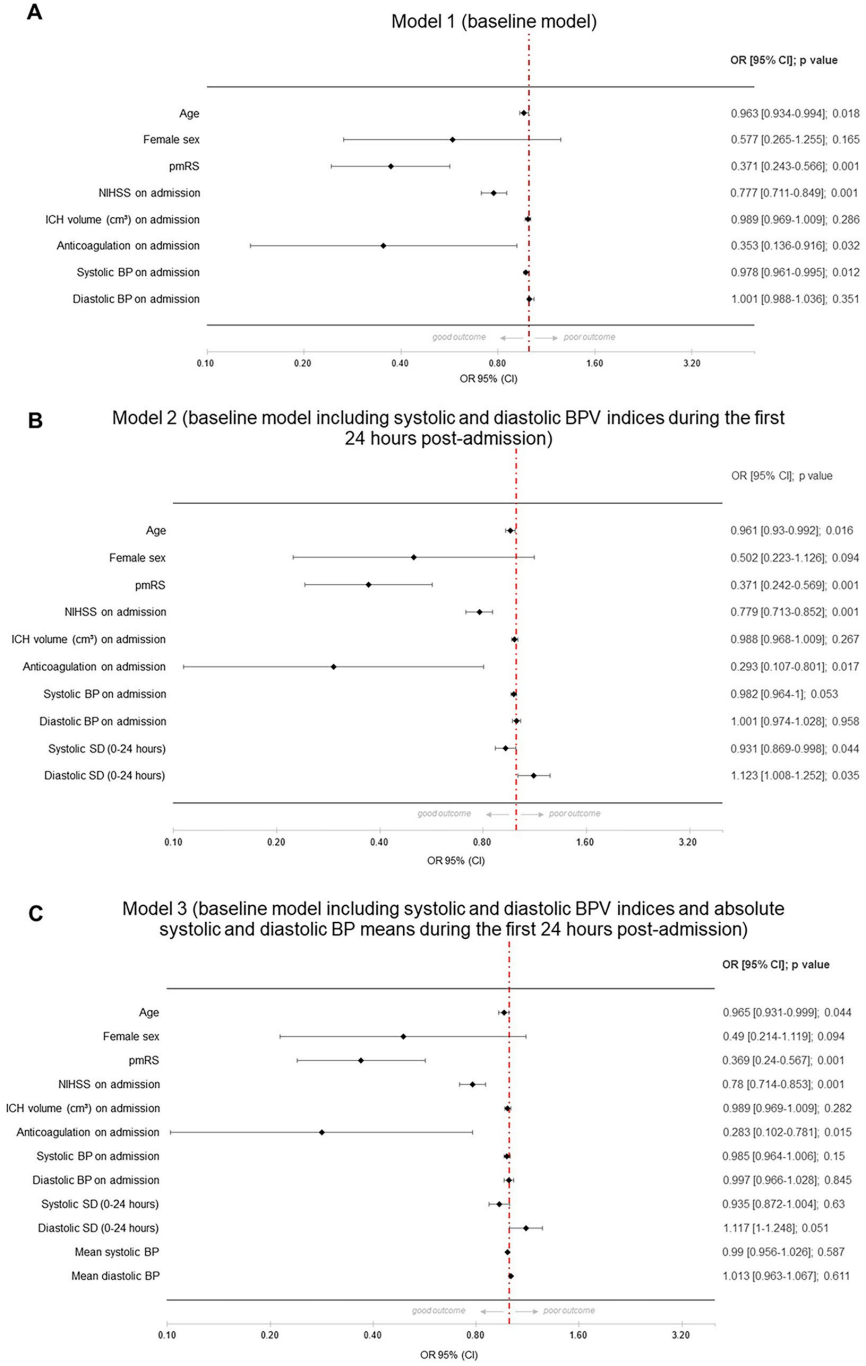
Forest plots with different models regarding good functional outcome after intracerebral hemorrhage (ICH)

**Fig. 2:**
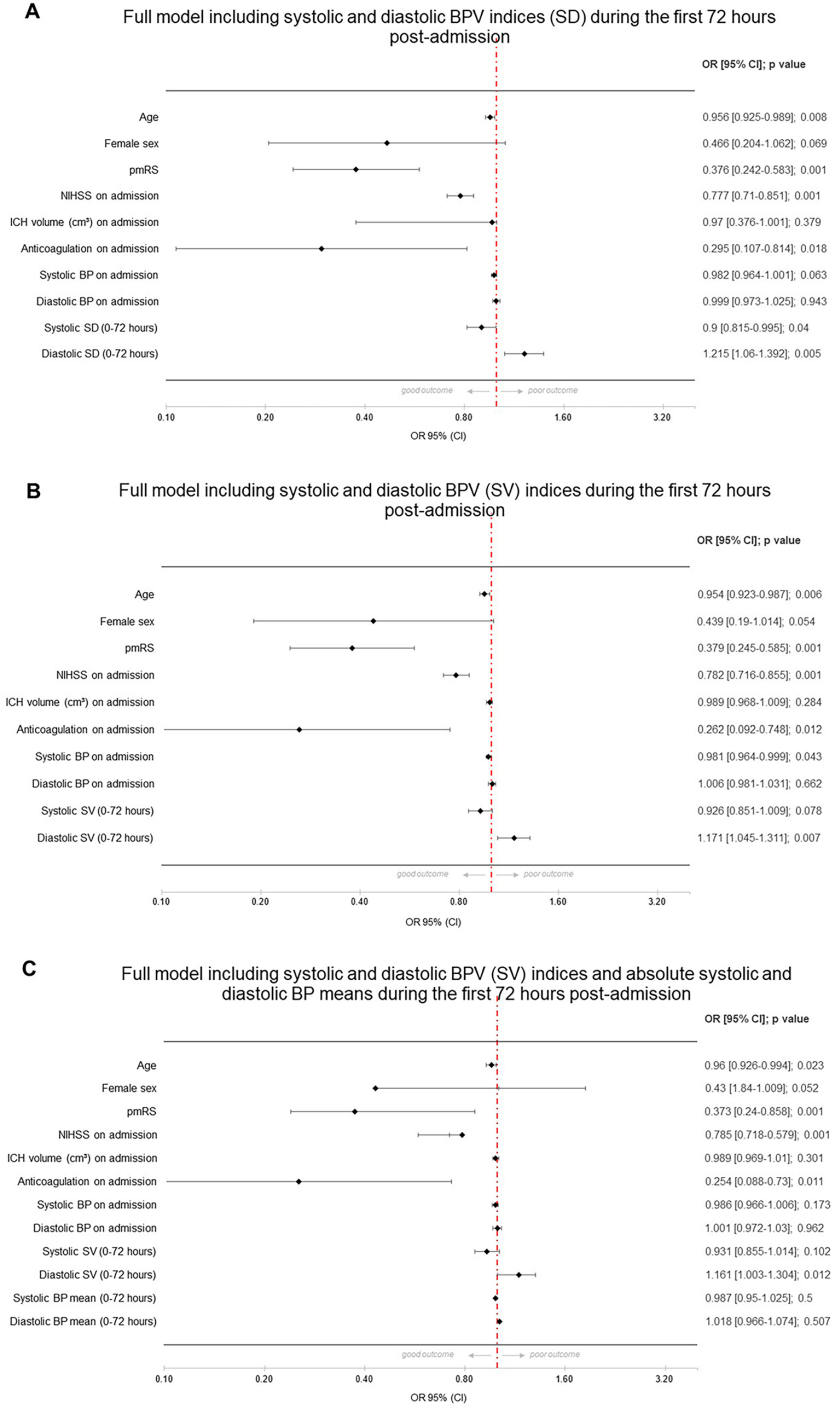

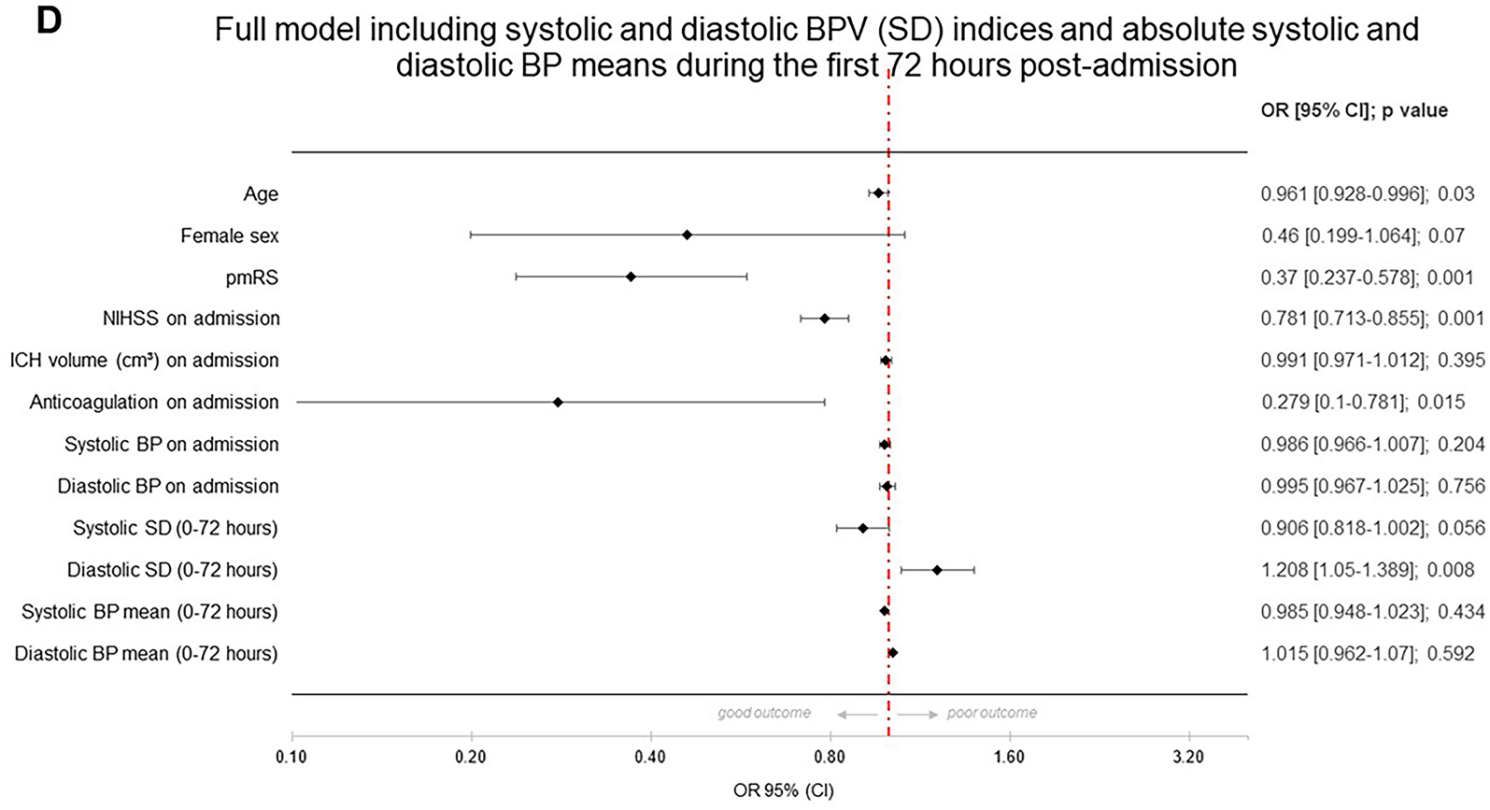
Forest plots with full model and regarding good functional outcome after intracerebral hemorrhage (ICH)

In the baseline model, significant predictors of good functional outcome were the NIHSS score on admission, the mRS score before the index event, and the presence of preanticoagulation treatment. These factors were associated with a decrease in the likelihood of a good outcome. Further, each unit increase in systolic BPV on admission was associated with a slight statistically significant decrease in the likelihood of good outcome (OR 0.978, *p* = 0.012) (see Fig. 1a). In model 2, adding different BPV indices for different time frames, these results persisted, whereas individual BPV indices (each systolic and diastolic) showed no significant effects, indicating that systolic and diastolic BPV are not individually. The only exemption was the model with diastolic BPV (SV) during 0–72 h (OR 1.092, 95% CI 1.008–1.184, *p* = 0.032). Clinically, this result suggested that a greater variability in diastolic BP within the first 0–72 h may be indicative of a more favorable prognosis. This could influence clinical decisions regarding the monitoring and management of BP in patients to optimize their chances of a better clinical outcome. Given the fact that individual BPV indices did not show convincing significant associations with good outcome in ICH, we decided to run a full model including systolic and diastolic BPV indices during the first 0–24 h after careful consideration. The SD of systolic BP over the first 24 h post ICH showed a negative association with good outcomes (OR 0.931, *p* = 0.044), and the SD of diastolic BP demonstrated a positive association (OR 1.123, *p* = 0.035), indicating that higher systolic variability is associated with a slight decrease in the likelihood of a good outcome, whereas higher diastolic variability appears to increase the likelihood slightly (see Fig. 1b). In a further analysis including mean systolic and diastolic BP during the first 24 h, systolic and diastolic BPV (OR 0.935 and 1.117, *p* = 0.063 and 0.051, respectively) showed a similar nonsignificant trend, whereas the absolute mean values of systolic and diastolic BP over the same period do not appear to be significant predictors of the clinical outcome in this analysis (see Fig. 1c). In further analysis for the time period 0–72 h (see Fig. 2a–d), systolic BPV regarding SD did not show a consistent or significant association with the likelihood of good clinical outcome. The *p* values across the models (0.040, 0.078, and 0.056) hovered around the threshold of significance, suggesting a potential trend but not a definitive conclusion. Diastolic BPV (SD) showed a positive and significant association with a good clinical outcome across all models, with ORs indicating an 11.7% to 20.8% increase in the likelihood of good outcome per unit increase in the SD. The consistency of statistical significance (*p* values of 0.005, 0.007, and 0.008) across the models strengthened the evidence that greater diastolic BPV is associated with better clinical outcomes. Mean systolic and diastolic BP showed no significant association with the outcome.

## Discussion

Our prospective observational study on a well-described cohort of patients with nontraumatic ICH revealed a nuanced relationship between patient-related risk factors as well as systolic and diastolic BPV over various time frames in the acute phase following ICH and patient outcome when comparing patients with good and poor functional outcome at follow-up after 3 months.

Firstly, our analysis highlighted the pivotal role of early systolic BP management. Patients with good outcome exhibited significantly lower systolic BP upon admission, a trend that persisted over the first 72 h, underscoring further the importance of maintaining systolic BP below the critical threshold of 140 mm Hg to optimize outcomes. The less frequently the critical threshold of 140 mm Hg was exceeded, the more likely a good outcome was achieved. This is in line with existing guidelines and previous studies advocating for early systolic BP control [2, 14]. Conversely, patients with good outcomes demonstrated higher diastolic BP, particularly in the early stages of intensive care. The association with higher diastolic BP, however, challenges the traditional emphasis on lowering BP indiscriminately and highlights the potential necessity of maintaining adequate diastolic perfusion pressure, which may be crucial for cerebral blood flow and tissue perfusion during the acute phase of ICH. When considering the cerebral blood flow, only MAP and systolic BP have been evaluated so far [34]. The clinical implication of this finding is significant and moreover challenging for the traditional clinical focus on systolic BP alone, suggesting that a balanced approach to BP management that considers both systolic and diastolic components may be beneficial. The pathophysiological basis for the association between higher diastolic BP and better outcomes in ICH is unclear. However, higher diastolic BP might help maintain cerebral perfusion pressure and might reflect better vascular compliance and autoregulation [35].

Secondly, our findings regarding BPV offer novel insights. Lower systolic BPV was consistently associated with good outcomes, reinforcing the detrimental impact of excessive systolic fluctuations on functional outcome. Paradoxically, higher diastolic BPV correlated with improved outcomes, implying that diastolic variability might reflect beneficial adaptive responses or preserved cerebral autoregulation capacity [36, 37]. The nuanced role of diastolic BPV underscores the complexity of cerebral hemodynamics post- CH and prompts a reevaluation of current BP management strategies.

Thirdly, our multivariate analysis underscored the independent prognostic value of diastolic BPV as a potential protective factor. Systolic BPV consistently showed a negative correlation with good outcomes, whereas diastolic BPV emerged as a potential protective factor. These results are challenging traditional views and are also supporting a more dynamic approach to BP management in acute ICH care. Quite a few studies have examined the role of systolic BPV on several ICH-related outcomes. High systolic BPV (with BP values obtained every 15 min over the first 24 h), expressed as SD, was associated with early neurological deterioration [20]. Higher SV and SD of systolic BPV (with BP values measured every 15 min during the first 2 h after initiation of antihypertensive therapy and every 60 min during the next 22, 48, and 72 h) were also associated with neurological deterioration [21]. Moreover, in a post hoc analysis of INTERACT-2, higher SD of systolic BP was associated with mortality at 90 days [23]. Higher SD and CV of systolic BP (with BP monitored every 15 min during the first 2 h) were significantly associated with poor clinical outcome (mRS score ≥ 3) at 90 days’ follow-up in patients with nonlobar ICH [22]. Further, in a secondary analysis of ATACH-2, increased BPV of systolic BP was associated with poor outcome at 90 days’ follow-up (defined as mRS score ≥ 3) [25].

In our study’s context, the approach of quantifying BPV merits discussion, as different methodologies can yield varied interpretations. BPV is a multifaceted parameter, and its measurement can be influenced by the definitions and indices used [17, 18]. Traditionally, BPV has been characterized by SD, which measures the dispersion of BP readings around the mean, offering a straightforward assessment of variability. However, SD does not account for the sequence of BP changes and may not fully capture the clinical impact of BP fluctuations [17, 18]. SV focuses on the changes between consecutive BP readings, capturing short-term fluctuations that could be pivotal in acute ICH management. SV can highlight the immediate responses in BP, which may reflect the dynamic nature of cerebrovascular regulation in the acute phase following an ICH event. The CV, a normalized measure of variability relative to the mean BP, provides insights that are independent of the absolute BP level. Although CV offers comparability across patient populations with different mean BPs, it could potentially obscure the absolute magnitude of variability, which might have distinct physiological implications in the setting of ICH. Conversely, our findings regarding diastolic BPV challenge this perspective, as higher diastolic variability was associated with better outcomes. This could reflect more than just a compensatory physiological response; it may represent a marker of cerebral autoregulatory reserve or the ability of cerebral vasculature to adapt to the acute stressors following ICH [38].

In our study, we could identify several patient-related factors and acute treatment interventions that were significantly associated with functional outcomes following ICH. The average profile of our patients with ICH –(predominantly hypertensive, with a considerable subset also having cardiovascular risk factors, including atrial fibrillation and ongoing anticoagulant therapy) reinforces the complex clinical backdrop against which acute ICH management occurs. Notably, younger age and lower initial ICH severity, as indicated by NIHSS and ICH scores, were associated with good outcomes at 90 days’ follow-up. These associations underscore the significance of baseline clinical severity and preexisting health conditions in the prognosis of ICH, aligning with existing research that highlights the impact of these factors on stroke recovery [39–41]. The observation that patients with good outcomes had smaller initial ICH volumes and a lower incidence of intraventricular hemorrhage adds to the growing body of evidence suggesting the importance of early hemorrhage containment in determining patient prognosis [42]. Although anticoagulation therapy is a known risk factor for ICH, our findings suggest that its impact on outcomes is nuanced, with a higher prevalence of anticoagulation use among patients with poor outcomes [43]. Our data also revealed that patients with unfavorable outcomes required more aggressive acute interventions and had longer ICU and hospital stays [39–41].

The major strength of our study is the continuous BP monitoring via an arterial line over the first 72 h post admission and the well-characterized ICH cohort. We attempted to minimize any latent cofounding effect of ICH outcome-related predictors, by including them in the regression models.

The observational nature of the study precludes the establishment of a causal relationship between BPV and ICH outcomes. Additionally, the exclusion criteria may have resulted in the omission of patients with more severe presentations, and therefore our cohort with a median ICH score of 1 and ICH volume of 17 mL was less sick, potentially influencing the generalizability of the results. As such, our results do not necessarily denote a direct causal effect of BPV on post-ICH mRS scores at discharge. BPV might represent a consequence rather than a predictor of ICH outcome. Alternatively, healthier individuals with better outcomes might have more stable BP and BPV. Because of the large number of statistical tests, there is the possibility of type I errors. Moreover, the volumes of the ICHs were determined exclusively using the ABC/2 method. However, it is known that the ABC/2 method, particularly with irregularly shaped hematomas, can either underestimate or overestimate the volume, leading to some uncertainty in the results [44]. Additionally, our follow-up period of 90 days may have been too short for the recovery of patients with ICH. Causality from this monocentric observational study with a sample size of 216 patients is challenging; therefore, these associations should be further explored in prospective controlled studies with at least a 12-month follow-up period.

## Conclusions

Our findings highlight the complex interplay of demographic, clinical, and treatment-related factors in determining outcomes after ICH. Our study showed the prognostic significance of BPV, particularly diastolic BPV, in the acute phase of ICH, advocating for the inclusion of BPV in future prospective studies on the effect of BP and BPV on complications and outcome following ICH. This study supports a nuanced approach to BP management, tailored to individual BPV patterns, and underscores the potential of leveraging electronic health records and artificial intelligence in optimizing BP in acute ICH care. Future research should aim to incorporate continuous monitoring of cerebral autoregulation to elucidate the mechanisms by which BPV influences ICH outcomes.

## Supplementary Information

Below is the link to the electronic supplementary material.

Supplementary file1 (JPG 11005 KB)

Supplementary file2 (DOCX 18 KB)

## References

[1] Rothwell PM, Coull AJ, Giles MF, Howard SC, Silver LE, Bull LM, et al. Change in stroke incidence, mortality, case-fatality, severity, and risk factors in Oxfordshire, UK from 1981 to 2004 (Oxford vascular study). Lancet. 2004;363(9425):1925–33. 15194251 10.1016/S0140-6736(04)16405-2

[2] Greenberg SM, Ziai WC, Cordonnier C, Dowlatshahi D, Francis B, Goldstein JN, et al. Guideline for the management of patients with spontaneous intracerebral hemorrhage: a guideline from the American heart association/American stroke association. Stroke. 2022;53(7):e282–361. 35579034 10.1161/STR.0000000000000407

[3] Ovbiagele B, Goldstein LB, Higashida RT, Howard VJ, Johnston SC, Khavjou OA, et al. Forecasting the future of stroke in the United States: a policy statement from the American heart association and American stroke association. Stroke. 2013;44(8):2361–75. 23697546 10.1161/STR.0b013e31829734f2

[4] Watson N, Bonsack F, Sukumari-Ramesh S. Intracerebral hemorrhage: the effects of aging on brain injury. Front Aging Neurosci. 2022;14:859067. 35547620 10.3389/fnagi.2022.859067 PMC9082316

[5] Gross BA, Jankowitz BT, Friedlander RM. Cerebral intraparenchymal hemorrhage: a review. JAMA. 2019;321(13):1295–303. 30938800 10.1001/jama.2019.2413

[6] Woo D, Comeau ME, Venema SU, Anderson CD, Flaherty M, Testai F, et al. Risk factors associated with mortality and neurologic disability after intracerebral hemorrhage in a racially and ethnically diverse cohort. JAMA Netw Open. 2022;5(3):e221103. 35289861 10.1001/jamanetworkopen.2022.1103 PMC8924717

[7] Stanton R, Demel SL, Flaherty ML, Antzoulatos E, Gilkerson LA, Osborne J, et al. Risk of intracerebral haemorrhage from hypertension is greatest at an early age. Eur Stroke J. 2021;6(1):28–35. 33817332 10.1177/2396987321994296 PMC7995317

[8] Naito H, Hosomi N, Kuzume D, Nezu T, Aoki S, Morimoto Y, et al. Increased blood pressure variability during the subacute phase of ischaemic stroke is associated with poor functional outcomes at 3 months. Sci Rep. 2020;10(1):811. 31964961 10.1038/s41598-020-57661-z PMC6972830

[9] Anderson CS, Heeley E, Huang Y, Wang J, Stapf C, Delcourt C, et al. Rapid blood-pressure lowering in patients with acute intracerebral hemorrhage. N Engl J Med. 2013;368(25):2355–65. 23713578 10.1056/NEJMoa1214609

[10] Qureshi AI, Palesch YY, Barsan WG, Hanley DF, Hsu CY, Martin RL, et al. Intensive blood-pressure lowering in patients with acute cerebral hemorrhage. N Engl J Med. 2016;375(11):1033–43. 27276234 10.1056/NEJMoa1603460 PMC5345109

[11] Frontera JA. Blood pressure in intracerebral hemorrhage–how low should we go? N Engl J Med. 2013;368(25):2426–7. 23713579 10.1056/NEJMe1305047

[12] Keller DL. Blood-pressure lowering in acute intracerebral hemorrhage. N Engl J Med. 2013;369(13):1273. 24066753 10.1056/NEJMc1309586

[13] Drawz PE, Ix JH. BP Measurement in clinical practice: time to SPRINT to guideline-recommended protocols. J Am Soc Nephrol. 2018;29(2):383–8. 29051347 10.1681/ASN.2017070753 PMC5791063

[14] Ma L, Hu X, Song L, Chen X, Ouyang M, Billot L, et al. The third intensive care bundle with blood pressure reduction in acute cerebral haemorrhage trial (INTERACT3): an international, stepped wedge cluster randomised controlled trial. Lancet. 2023;402(10395):27–40. 37245517 10.1016/S0140-6736(23)00806-1 PMC10401723

[15] Hawkes MA, Anderson CS, Rabinstein AA. Blood pressure variability after cerebrovascular events: a possible new therapeutic target: a narrative review. Neurology. 2022;99(4):150–60. 35879090 10.1212/WNL.0000000000200856

[16] Andalib S, Lattanzi S, Di Napoli M, Petersen A, Biller J, Kulik T, et al. Blood pressure variability: a new predicting factor for clinical outcomes of intracerebral hemorrhage. J Stroke Cerebrovasc Dis. 2020;29(12):105340. 33017754 10.1016/j.jstrokecerebrovasdis.2020.105340

[17] Stevens SL, Wood S, Koshiaris C, Law K, Glasziou P, Stevens RJ, et al. Blood pressure variability and cardiovascular disease: systematic review and meta-analysis. BMJ. 2016;354:i4098. 27511067 10.1136/bmj.i4098 PMC4979357

[18] Parati G, Ochoa JE, Lombardi C, Bilo G. Assessment and management of blood-pressure variability. Nat Rev Cardiol. 2013;10(3):143–55. 23399972 10.1038/nrcardio.2013.1

[19] Liu W, Zhuang X, Zhang L. Prognostic value of blood pressure variability for patients with acute or subacute intracerebral hemorrhage: a meta-analysis of prospective studies. Front Neurol. 2021;12:606594. 33776881 10.3389/fneur.2021.606594 PMC7991598

[20] Rodriguez-Luna D, Pineiro S, Rubiera M, Ribo M, Coscojuela P, Pagola J, et al. Impact of blood pressure changes and course on hematoma growth in acute intracerebral hemorrhage. Eur J Neurol. 2013;20(9):1277–83. 23647568 10.1111/ene.12180

[21] Tanaka E, Koga M, Kobayashi J, Kario K, Kamiyama K, Furui E, et al. Blood pressure variability on antihypertensive therapy in acute intracerebral hemorrhage: the stroke acute management with urgent risk-factor assessment and improvement-intracerebral hemorrhage study. Stroke. 2014;45(8):2275–9. 24968929 10.1161/STROKEAHA.114.005420

[22] Jeon JP, Kim C, Kim SE. Blood pressure variability and outcome in patients with acute nonlobar intracerebral hemorrhage following intensive antihypertensive treatment. Chin Med J (Engl). 2018;131(6):657–64. 29521287 10.4103/0366-6999.226886 PMC5865310

[23] Manning L, Hirakawa Y, Arima H, Wang X, Chalmers J, Wang J, et al. Blood pressure variability and outcome after acute intracerebral haemorrhage: a post-hoc analysis of INTERACT2, a randomised controlled trial. Lancet Neurol. 2014;13(4):364–73. 24530176 10.1016/S1474-4422(14)70018-3

[24] Chung PW, Kim JT, Sanossian N, Starkmann S, Hamilton S, Gornbein J, et al. Association between hyperacute stage blood pressure variability and outcome in patients with spontaneous intracerebral hemorrhage. Stroke. 2018;49(2):348–54. 29301973 10.1161/STROKEAHA.117.017701

[25] de Havenon A, Majersik JJ, Stoddard G, Wong KH, McNally JS, Smith AG, et al. Increased blood pressure variability contributes to worse outcome after intracerebral hemorrhage. Stroke. 2018;49(8):1981–4. 30012822 10.1161/STROKEAHA.118.022133 PMC6202185

[26] Meeks JR, Bambhroliya AB, Meyer EG, Slaughter KB, Fraher CJ, Sharrief AZ, et al. High in-hospital blood pressure variability and severe disability or death in primary intracerebral hemorrhage patients. Int J Stroke. 2019;14(9):987–95. 30681042 10.1177/1747493019827763

[27] Moullaali TJ, Wang X, Martin RH, Shipes VB, Robinson TG, Chalmers J, et al. Blood pressure control and clinical outcomes in acute intracerebral haemorrhage: a preplanned pooled analysis of individual participant data. Lancet Neurol. 2019;18(9):857–64. 31397290 10.1016/S1474-4422(19)30196-6

[28] Stulberg EL, Harris BRE, Zheutlin AR, Delic A, Sheibani N, Anadani M, et al. Association of blood pressure variability with death and discharge destination among critically ill patients with and without stroke. Neurology. 2023;101(11):e1145–57. 37487742 10.1212/WNL.0000000000207599 PMC10513881

[29] Raposo N, Zanon Zotin MC, Seiffge DJ, Li Q, Goeldlin MB, Charidimou A, et al. A causal classification system for intracerebral hemorrhage subtypes. Ann Neurol. 2023;93(1):16–28. 36197294 10.1002/ana.26519 PMC9839566

[30] Saver JL, Chaisinanunkul N, Campbell BCV, Grotta JC, Hill MD, Khatri P, et al. Standardized nomenclature for modified Rankin scale global disability outcomes: consensus recommendations from stroke therapy academic industry roundtable XI. Stroke. 2021;52(9):3054–62. 34320814 10.1161/STROKEAHA.121.034480

[31] Fazekas F, Chawluk JB, Alavi A, Hurtig HI, Zimmerman RA. MR signal abnormalities at 1.5 T in Alzheimer’s dementia and normal aging. AJR Am J Roentgenol. 1987;149(2):351–6. 3496763 10.2214/ajr.149.2.351

[32] Mayer SA, Brun NC, Begtrup K, Broderick J, Davis S, Diringer MN, et al. Efficacy and safety of recombinant activated factor VII for acute intracerebral hemorrhage. N Engl J Med. 2008;358(20):2127–37. 18480205 10.1056/NEJMoa0707534

[33] Mayer SA, Brun NC, Begtrup K, Broderick J, Davis S, Diringer MN, et al. Recombinant activated factor VII for acute intracerebral hemorrhage. N Engl J Med. 2005;352(8):777–85. 15728810 10.1056/NEJMoa042991

[34] Singh V, Cheng R. Chapter 5—neurovascular physiology and neurocritical care. In: Hetts SW, Cooke DL, editors. Handbook of Clinical Neurology, vol. 176. Amsterdam: Elsevier; 2021. p. 71–80. 10.1016/B978-0-444-64034-5.00014-6 33272411

[35] Beqiri E, Garcia-Orellana M, Politi A, Zeiler FA, Placek MM, Fabregas N, et al. Cerebral autoregulation derived blood pressure targets in elective neurosurgery. J Clin Monit Comput. 2024;38(3):649–62. 38238636 10.1007/s10877-023-01115-0 PMC11164832

[36] Aaslid R, Lindegaard KF, Sorteberg W, Nornes H. Cerebral autoregulation dynamics in humans. Stroke. 1989;20(1):45–52. 2492126 10.1161/01.str.20.1.45

[37] Armstead WM. Cerebral blood flow autoregulation and dysautoregulation. Anesthesiol Clin. 2016;34(3):465–77. 27521192 10.1016/j.anclin.2016.04.002 PMC4988341

[38] Silverman A, Petersen NH. Physiology, cerebral autoregulation. In: StatPearls. Treasure Island (FL): StatPearls Publishing Copyright © 2024, StatPearls Publishing LLC; 2024. 31985976

[39] Widyadharma IPE, Krishna A, Soejitno A, Laksmidewi A, Tini K, Putra IBK, et al. Modified ICH score was superior to original ICH score for assessment of 30-day mortality and good outcome of non-traumatic intracerebral hemorrhage. Clin Neurol Neurosurg. 2021;209:106913. 34507127 10.1016/j.clineuro.2021.106913

[40] Sembill JA, Gerner ST, Volbers B, Bobinger T, Lücking H, Kloska SP, et al. Severity assessment in maximally treated ICH patients: the max-ICH score. Neurology. 2017;89(5):423–31. 28679602 10.1212/WNL.0000000000004174

[41] Sembill JA, Castello JP, Sprügel MI, Gerner ST, Hoelter P, Lücking H, et al. Multicenter validation of the max-ICH score in intracerebral hemorrhage. Ann Neurol. 2021;89(3):474–84. 33222266 10.1002/ana.25969

[42] Panchal HN, Shah MS, Shah DS. Intracerebral hemorrhage score and volume as an independent predictor of mortality in primary intracerebral hemorrhage patients. Indian J Surg. 2015;77(Suppl 2):302–4. 26730014 10.1007/s12262-012-0803-2 PMC4692889

[43] Wang X, Wen D, Chen Y, You C, Ma L. Anticoagulation medication in nontraumatic intracranial hemorrhage survivors with atrial fibrillation. J Thromb Thrombolysis. 2023;56(1):1–11. 37022508 10.1007/s11239-023-02804-y

[44] Divani AA, Majidi S, Luo X, Souslian FG, Zhang J, Abosch A, et al. The ABCs of accurate volumetric measurement of cerebral hematoma. Stroke. 2011;42(6):1569–74. 21566231 10.1161/STROKEAHA.110.607861

