# Supplementary figures and images for: Continuous Blood Pressure Indices During the First 72 Hours and Functional Outcome in Patients with Spontaneous Intracerebral Hemorrhage
Source: Neurocrit Care. 2024 Oct 25;42(3):839–56. doi: 10.1007/s12028-024-02146-4 (PMC12137446; doi:10.1007/s12028-024-02146-4)

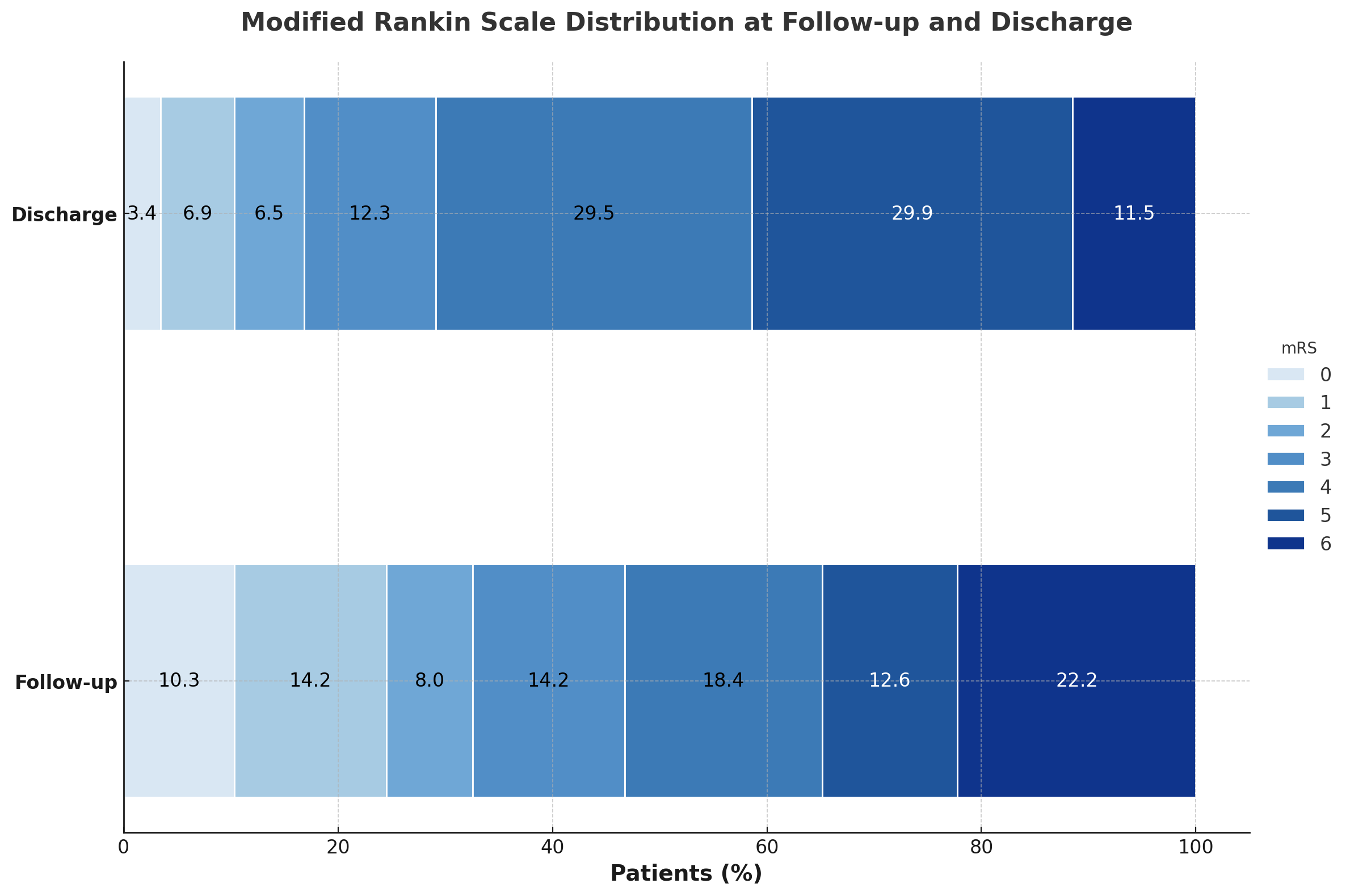

Supplement: Supplementary file 1 — Supplementary file1 (JPG 11005 KB) [file 12028_2024_2146_MOESM1_ESM.jpg]
