# Supplementary material for: Continuous Blood Pressure Indices During the First 72 Hours and Functional Outcome in Patients with Spontaneous Intracerebral Hemorrhage
Source: Neurocrit Care. 2024 Oct 25;42(3):839–56. doi: 10.1007/s12028-024-02146-4 (PMC12137446; doi:10.1007/s12028-024-02146-4)
Supplement: Supplementary file 2 — Supplementary file2 (DOCX 18 KB) [file 12028_2024_2146_MOESM2_ESM.docx]

**Supplement material:**

**Supplement Table 1.** Descriptive comparison of absolute blood pressure in patients with ICH, based on good functional outcome (mRS 0-2) at 90 days follow-up: time frame 16-24hrs, 24-48hrs, 48-72hrs

|  | All ICH patients  n=305 |  | ICH patients with good outcome  n=106 | ICH patients with poor outcome  n=155 | P-value |
| --- | --- | --- | --- | --- | --- |
| **Time frame 16-24 h** |  |  |  |  |  |
| systolic BP mean±SD (mmHg) | 138.8±14.6 |  | 136.0±16.0 | 140.7±13.3 | 0.014 |
| Proportion of values exceeding 140 mmHg systolic (%)* | 0.48±0.32 |  | 0.45±0.34 | 0.51±0.32 | 0.129 |
| Diastolic BP mean±SD (mmHg) | 67.0±11,0 |  | 69.0±11.2 | 65.7±10.6 | 0.020 |
| MAP mean±SD (mmHg) | 93.3±11.2 |  | 94.0±12.0 | 92.8±10.7 | 0.399 |
| **Time frame 24-48 h** |  |  |  |  |  |
| systolic BP mean±SD (mmHg) | 140.1±12.9 |  | 137.1±13.3 | 142.0±12.4 | 0.004 |
| Proportion of values exceeding 140 mmHg systolic (%)* | 0.51±0.25 |  | 0.44±0.27 | 0.55±0.23 | <0.001 |
| Diastolic BP mean±SD (mmHg) | 67.4±10.2 |  | 69.4±10.8 | 66.0±9.7 | 0.010 |
| MAP mean±SD (mmHg) | 94.3±9.8 |  | 95.0±10.1 | 93.9±9.5 | 0.376 |
| **Time frame 48-72 h** |  |  |  |  |  |
| systolic BP mean±SD (mmHg) | 142.6±13.4 |  | 141.0±14.9 | 143.6±12.5 | 0.176 |
| Proportion of values exceeding 140 mmHg systolic (%)* | 0.56±0.26 |  | 0.51±0.29 | 0.59v±0.23 | 0.021 |
| Diastolic BP mean±SD (mmHg) | 68.8±10.6 |  | 72.4±10.2 | 66.8±10.4 | <0.001 |
| MAP mean±SD (mmHg) | 96.0±10.3 |  | 98.0±10.4 | 94.8±10.1 | 0.028 |

^Mann-Whitney U test for non normally or ordinal variables

*Chi-square test for normally distributed and continuous variables

Statistically significant values are highlighted

Abbreviations: BP, blood pressure; HR, heart rate; MAP, middle arterial pressure; SD, standard deviation.

* Percent values are represented as decimal numbers. For example, 0.25 means that 25% of the values are above the specified threshold.

Supplement table 2: Descriptive comparison of blood pressure variability indices in patients with ICH, based on good functional outcome (mRS 0-2) at 90 days follow-up: time frame 16-24hrs, 24-48hrs, 48-72hrs

|  | All ICH patients  n=305 |  | ICH patients with good outcome  n=106 | ICH patients with poor outcome  n=155 | P-value |
| --- | --- | --- | --- | --- | --- |
| **Time frame 8-16 h** |  |  |  |  |  |
| Systolic BP SV mean±SD (mmHg) | 14.3±8.4 |  | 12.5±7.1 | 15.5±9.0 | 0.005 |
| Systolic BP SD mean±SD (mmHg) | 14.1±6.9 |  | 12.0±6.2 | 15.6±7.0 | <0.001 |
| Systolic BP CV, Mean±SD* | 0.10±0.05 |  | 0.09±0.04 | 0.11±0.05 | <0.001 |
| Diastolic BP SV  mean±SD (mmHg) | 8.2±5.5 |  | 7.3±3.7 | 8.7±6.3 | 0.036 |
| Diastolic BP SD mean±SD (mmHg) | 7.7±4.4 |  | 6.9±2.3 | 8.3±5.2 | 0.011 |
| diastolic BP CV, Mean±SD* | 0.12±0.07 |  | 0.10±0.04 | 0.13±0.08 | 0.002 |
| MAP SV  mean±SD (mmHg) | 10.8±7.7 |  | 9.5±5.9 | 11.7±8.6 | 0.029 |
| MAP SD mean±SD (mmHg) | 10.6±6.5 |  | 9.3±5.2 | 11.4±7.1 | 0.011 |
| MAP CV, Mean±SD* | 0.11±0.07 |  | 0.10±0.05 | 0.13±0.07 | 0.003 |
| **Time frame 16-24 h** |  |  |  |  |  |
| Systolic BP SV mean±SD (mmHg) | 13.8±7.1 |  | 13.3±6.1 | 14.2±7.6 | 0.298 |
| Systolic BP SD mean±SD (mmHg) | 13.8±6.0 |  | 12.9±5.5 | 14.4±6.2 | 0.041 |
| Systolic BP CV, Mean±SD* | 0.10±0.05 |  | 0.10±0.04 | 0.10±0.05 | 0.161 |
| Diastolic BP SV  mean±SD (mmHg) | 8.3±4.9 |  | 8.7±5.1 | 8.0±4.7 | 0.285 |
| Diastolic BP SD mean±SD (mmHg) | 8.0±4.0 |  | 8.3±3.9 | 7.7±4.1 | 0.261 |
| diastolic BP CV, Mean±SD* | 0.12±0.06 |  | 0.13±0.06 | 0.12±0.06 | 0.545 |
| MAP SV  mean±SD (mmHg) | 10,5±6.1 |  | 10.8±6.9 | 10.2±5.4 | 0.498 |
| MAP SD mean±SD (mmHg) | 10.2±4.7 |  | 10.2±4.9 | 10.2±4.5 | 0.892 |
| MAP CV, Mean±SD* | 0.11±0.05 |  | 0.11±0.06 | 0.11±0.05 | 0.963 |
| **Time frame 24-48 h** |  |  |  |  |  |
| Systolic BP SV mean±SD (mmHg) | 14.9±6.1 |  | 14.7±5.9 | 15.0±6.2 | 0.713 |
| Systolic BP SD mean±SD (mmHg) | 16.4±5.1 |  | 15.7±5.2 | 17.0±5.1 | 0.066 |
| Systolic BP CV, Mean±SD* | 0.12±0.04 |  | 0.11±0.04 | 0.12±0.03 | 0.266 |
| Diastolic BP SV  mean±SD (mmHg) | 9.9±5.1 |  | 10.6±5.3 | 9.4±4.8 | 0.073 |
| Diastolic BP SD mean±SD (mmHg) | 9.7±3.9 |  | 10.3±4.4 | 9.9±3.9 | 0.084 |
| diastolic BP CV, Mean±SD* | 0.15±0.06 |  | 0.15±0.07 | 0.14±0.05 | 0.262 |
| MAP SV  mean±SD (mmHg) | 12.0±5.9 |  | 12.3±6.4 | 11.9±5.6 | 0.606 |
| MAP SD mean±SD (mmHg) | 12.5±4.8 |  | 12.4±5.2 | 12.9±5.0 | 0.793 |
| MAP CV, Mean±SD* | 0.13±0.05 |  | 0.13±0.06 | 0.14±0.04 | 0.728 |
| **Time frame 48-72 h** |  |  |  |  |  |
| Systolic BP SV mean±SD (mmHg) | 15.2±5.8 |  | 15.2±4.7 | 15.3±6.3 | 0.886 |
| Systolic BP SD mean±SD (mmHg) | 16.5±4.9 |  | 15.8±4.7 | 17.0±5.1 | 0.080 |
| Systolic BP CV, Mean±SD* | 0.12±0.04 |  | 0.11±0.03 | 0.12±0.04 | 0.150 |
| Diastolic BP SV  mean±SD (mmHg) | 10.2±5.1 |  | 11.2±5.4 | 9.6±4.8 | 0.024 |
| Diastolic BP SD mean±SD (mmHg) | 10.1±3.9 |  | 10.4±3.8 | 9.9±3.9 | 0.793 |
| diastolic BP CV, Mean±SD* | 0.15±0.06 |  | 0.14±0.05 | 0.15±0.06 | 0.453 |
| MAP SV  mean±SD (mmHg) | 12.0±5.0 |  | 12.5±4.8 | 11.6±5.1 | 0.255 |
| MAP SD mean±SD (mmHg) | 12.7±4.6 |  | 12.3±3.8 | 12.9±5.0 | 0.376 |
| MAP CV, Mean±SD* | 0.13±0.05 |  | 0.13±0.04 | 0.14±0.05 | 0.126 |

^Mann-Whitney U test for non normally or ordinal variables

*Chi-square test for normally distributed and continuous variables

Statistically significant values are highlighted

Abbreviations: BP, blood pressure; CV, coefficient of variation; MAP, middle arterial pressure; SD, standard deviation; SV, successive variability.

*Percent values are represented as decimal numbers. For example, 0.25 means that 25% of the values are above the specified threshold.
